# Insights into Formation of Bicontinuous Emulsion Gels via In Situ (Ultra-)Small Angle X‑Ray Scattering

**DOI:** 10.1021/acs.jpcb.5c02375

**Published:** 2025-06-12

**Authors:** Meyer T. Alting, Dominique M. E. Thies-Weesie, Alexander M. van Silfhout, Mariska de Ruiter, Theyencheri Narayanan, Martin F. Haase, Andrei V. Petukhov

**Affiliations:** † Van ‘t Hoff Laboratory for Physical and Colloid Chemistry, Department of Chemistry, Debye Institute for Nanomaterials Science, 3536 Utrecht University, Padualaan 8, Utrecht 3584CH, The Netherlands; ‡ 55553 ESRF–The European Synchrotron, 71 Avenue des Martyrs, Grenoble 38043, France

## Abstract

Nanostructured materials formed via kinetically controlled self-assembly processes gather more interest nowadays. In particular, bicontinuous emulsion gels stabilized by colloidal particles, called bijels, are attractive materials as they combine bulk properties of two immiscible liquids into an interwoven network structure. The limited understanding of the complex formation phenomena of bijels restricts the control over the synthesis, and so its applicability. In this work, in situ (ultra-) small-angle X-ray scattering is applied to gain insight into the phase separation and self-assembly kinetics of bijels formed via solvent transfer induced phase separation. An X-ray compatible microfluidic setup allows accessing the kinetics of the extrusion process with a millisecond resolution. The formation of such bijels out of a liquid precursor mixture is shown to occur via three consecutive steps. The first 7 ms of the extrusion are dominated by fluid dynamics. Then, the precursor mixture remains in an induction phase for 50 ms where nanoparticles start to self-assemble without structural development on the (sub)­micron scale. From 50 ms on, an inward propagation of a liquid–liquid phase separation front occurs, besides the proceeding nanoparticle self-assembly obtaining (sub)­micron-sized structures. This time-resolved monitoring technique offers valuable insights into the structural evolution of kinetically controlled materials and enhances our understanding of the formation of bicontinuous emulsion gels.

## Graphical abstract

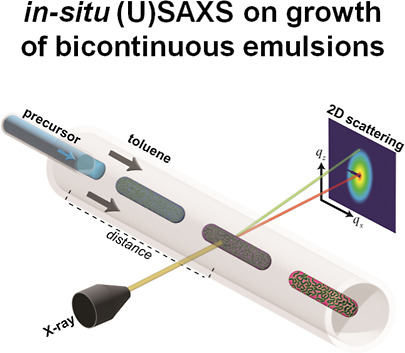

## 1 Introduction

Kinetically controlled self-assembly of nanostructured materials is an active research field due to its diverse applicability and complex formation mechanisms.^1–4^ Since two decades, fluid bicontinuous emulsions gels (bijels) make their advance in this field of self-assembly.^5–7^ Bijels consist of two immiscible liquids that are mechanically arrested in a nonequilibrium, bicontinuous arrangement by a percolating layer of colloids at the liquid–liquid interface. These unique structures have high potential in areas like catalysis,^8,9^ separation membranes,^10^ biomaterials^11,12^ and energy storage.^13,14^ Limited control over the formation mechanisms of bijels, however, restricts their applicability.

The formation of bijels is a complex, nonequilibrium self-assembly process.^7,15^ Generally, bijels are formed by inducing phase separation of a liquid mixture containing surface-active colloidal particles into two immiscible liquids via spinodal decomposition.^6,16–20^ The particles self-assemble on the interface between both interwoven liquids to minimize the interfacial area. Eventually, the particles form a dense layer around the liquid domains that kinetically arrests the liquids from further thermodynamically driven demixing. The obtained structure forms a viscoelastic bicontinuous emulsion with fully interconnected liquid domains.^21^

The obtained structure of the bijel sensitively depends on many factors, such as the exact formulation of the precursor mixture, type of colloidal particles and their surface chemistry, temperature and fabrication method.^14,19,22–24^ To the best of our knowledge, studies on the influence of these factors are based on ex situ characterization of bijel structures after completing phase separation. The phenomena occurring during spinodal decomposition, such as the self-assembly of particles, have not yet been extensively investigated in situ due to common limitations in microscopy like optical transparency or lack of contrast.^6^ Prior research on bijel-templated membranes proposed a method to study bijel formation by polymerizing intermediate phase separated stages.^10,25^ Since this method requires polymerization of one liquid phase, its applicability is restricted to such polymerizable bicontinuous emulsions. The complex control over the formation of bijels consisting of two liquid phases calls for new in situ methods to study the phase separation and consecutive self-assembly of particles during phase separation.

Here, we present a microfluidic setup suitable for synchrotron small-angle X-ray scattering (SAXS) to investigate the dynamic self-assembly of colloids during bijel formation in real time. The advantage of SAXS over microscopy techniques is that it neither requires optical transparency nor changes to experimental conditions: nearly all nanoparticle and colloidal systems in both soft- and hard matter can be probed.^26–31^ In situ SAXS has already been successfully employed in monitoring self-assembly behavior of nanoparticles in the formation of e.g. emulsions, mesoporous materials and nanostructures.^32–35^ Traditional SAXS gives access to structural features on the nanometer scale and is suitable to observe primary particles and their positional correlations with neighboring particles during phase separation (∼10^0^–10^2^ nm). Extending the technique to ultrasmall angles (USAXS) enables analysis on larger scales (∼10^2^–10^3^ nm) to shed light onto larger features formed during phase separation, like aggregates and pores.^27,36,37^

In this work, we investigate the kinetics of phase separation and self-assembly of nanoparticles during bijel formation. We perform in situ SAXS measurements during the synthesis of pinched-off bijel fragments fabricated out of a critical precursor mixture undergoing spinodal decomposition via solvent transfer induced phase separation (STrIPS) in a microfluidic device. SAXS analysis reveals that individual nanoparticles become more attractive and self-assemble at the liquid–liquid interface during phase separation. We propose a two-phase model to simulate the attachment of the particles onto the interface. Furthermore, we show that USAXS probes the phase separation itself and the formation of the tortuous structure. In addition, the combination of SAXS and USAXS reveals that the local particle self-assembly occurs significantly earlier than the formation of (sub)­micron sized structures. These results show that the self-assembly of the particles and the formation of the tortuous bijel network can be followed in real time. This time-resolved methodology can be extended to other fabrication methods and critical mixtures to obtain a deeper understanding of the kinetic pathways involved during bijel synthesis.

## 2 Method Section

### 2.1 Materials

All chemicals were used as received. Diethyl phthalate (DEP, 99%), glycerol (99+%, synthetic), hydrochloric acid (37%) and toluene (99+%, extra pure) were received from Thermo Scientific. Hexadecyltrimethylammonium bromide (CTAB, ≥99%), light mineral oil (density 0.84 g mL^–1^) and 1-propanol (≥99.5%) were purchased from Sigma-Aldrich. Silica nanoparticles (Ludox TMA, batch number 1003481587, particle diameter 29 nm) were obtained from Grace GmbH. *n*-Hexane (99%, HPLC) was received from Biosolve BV. Octadecyl trichlorosilane (OTS, 94.3%) was purchased from Santa Cruz Biotechnology, Inc. Water used in all experiments is ultrapure Milli-Q water purified by a Rephile Genie U2 system with a resistivity of 18.2 MΩ·cm.

### 2.2 Preparation of Bijel Precursor

100 g of 34 wt % Ludox TMA dispersion (batch number 1003481587) is concentrated from 34 wt % to 52 wt % in a rotary evaporator (Heidolph Instruments) at 60 °C and 140 mbar. The dispersion is centrifuged at 3750 g for 15 min (Allegra X–12R, Beckman Coulter) to remove any particle aggregates present. The weight percentage was determined by evaporation of 2 mL supernatant and consequent dry mass determination. Then, the dispersion was diluted to 50 wt % by adding Milli-Q water. The particles were acidified to pH 1.8 using 1 M aqueous HCl shortly before adding to the precursor as described below.

The bijel precursor mixture consists of four liquids with weight fractions *w*_DEP_ = 0.084, $w_{H_{2}O}$ = 0.423, *w*_glycerol_ = 0.121 and *w*_1‑propanol_ = 0.372. In addition, the mixture contains Ludox TMA nanoparticles with a weight fraction of 0.301 and a CTAB concentration of 28.2 mM with respect to, respectively, the total mass and volume of the four liquids. Thirteen mL precursor is prepared by mixing the following liquids: 0.853 g DEP, 1.209 g of 200 mM CTAB in 1-propanol, 2.446 g of 50 wt % glycerol in 1-propanol, 1.338 g 1-propanol and 8.650 g of the 50 wt % Ludox TMA dispersion as discussed above. Additional details about the preparation of these bijel precursor mixtures can be found in ref 38 and .^39^

### 2.3 Assembly of Microfluidic Device

All borosilicate glass capillaries are purchased from CM Scientific. A round capillary (inner diameter (ID) 50 μm, outer diameter (OD) 80 μm, CV0508) is glued in a square capillary (ID 100 μm, OD 200 μm, 8510–050) with Norland adhesive 81. This assembly is inserted into a round capillary (ID 300 μm, OD 400 μm, CV3040) and centered. The ends of the square- and outer round capillaries are glued on separate microscopy slides using epoxy glue (Liqui Moly 6183). The assembly is glued on a custom-made holder leaving the capillaries uncovered (Figure 1). Needles as flushing- and outlet channel are glued, respectively, near the 50 μm capillary and on the end of the outer capillary. Two dispensing needles are glued above the openings of both round capillaries as inlets. The needle-assembly is embedded in epoxy glue to prevent leakage.

**Figure 1:**
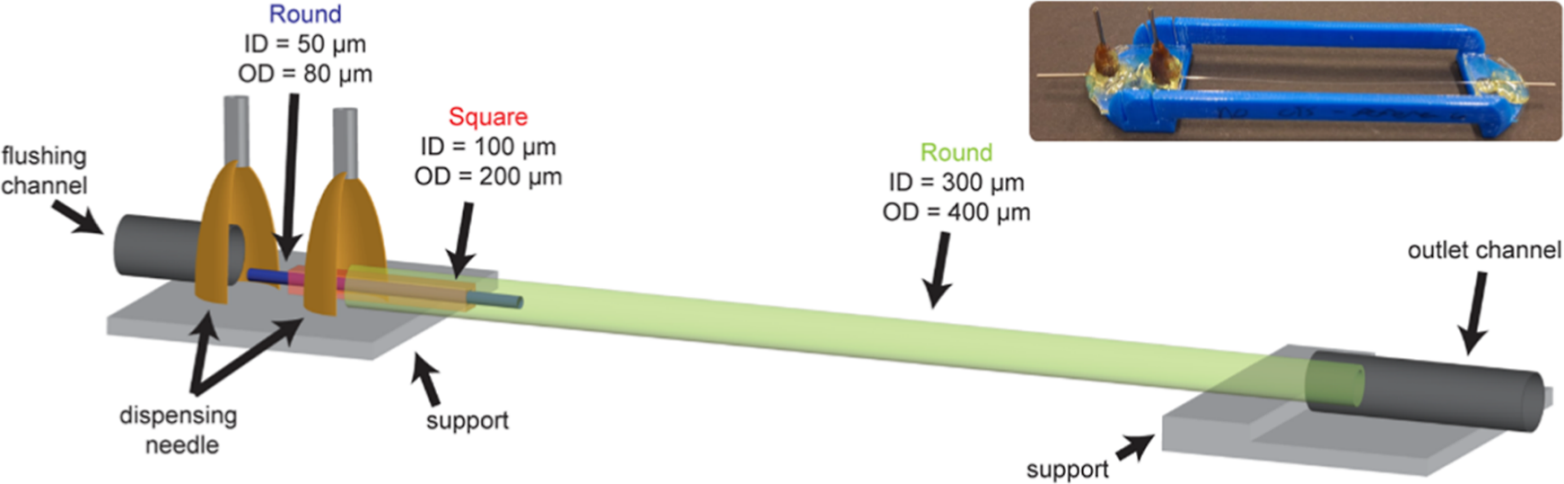
Schematic representation of microfluidic device for extruding bijel fragments. Photograph shows an actual device placed in a custom-made holder.

### 2.4 Microfluidic Device Hydrophobization

A microfluidic device was initially filled with 9 M aqueous KOH/NaOH solution for 6 h and rinsed with water. Then, 3 vol % OTS solution in mineral oil was filled in the capillaries, heated for 2 min using hot air and rinsed with *n*-hexane.

### 2.5 Enrichment of Toluene by Water

Toluene was enriched with water by vigorously shaking 250 mL toluene with 20 mL Milli-Q water for 5 min at room temperature. The mixture phase separated for 1 h prior using the top-layer of the oil-phase.

### 2.6 Bijel Fragment Extrusion in Microfluidic Device in SAXS Beamline

Three mL precursor mixture was loaded in a 5 mL syringe (BD Discardit, Ø12.40 mm) and 20 mL water-enriched toluene in a 20 mL syringe (BD Discardit, Ø20.00 mm). The syringes are connected to the inlets of the microfluidic device using PTFE tubing (Cole-Parmer Instrument Company). The outlet of the device is connected to a 100 mL collection vial using the same tubing. The syringes are placed in two syringe pumps (World Precision Instruments, model AL-1010). The microfluidic device is positioned horizontally on a movable stage in the beamline as depicted in Figure 2D in the main text. The precursor mixture flows with a flow rate of 0.40 mL h^–1^ and, simultaneously, water-enriched toluene with a flow rate of 17.50 mL h^–1^ through the microfluidic device. 50 μm diameter 5 mm long bijel fragments were extruded at ∼30 s^–1^ with a spacing of 3–5 mm between the fragments.

**Figure 2:**
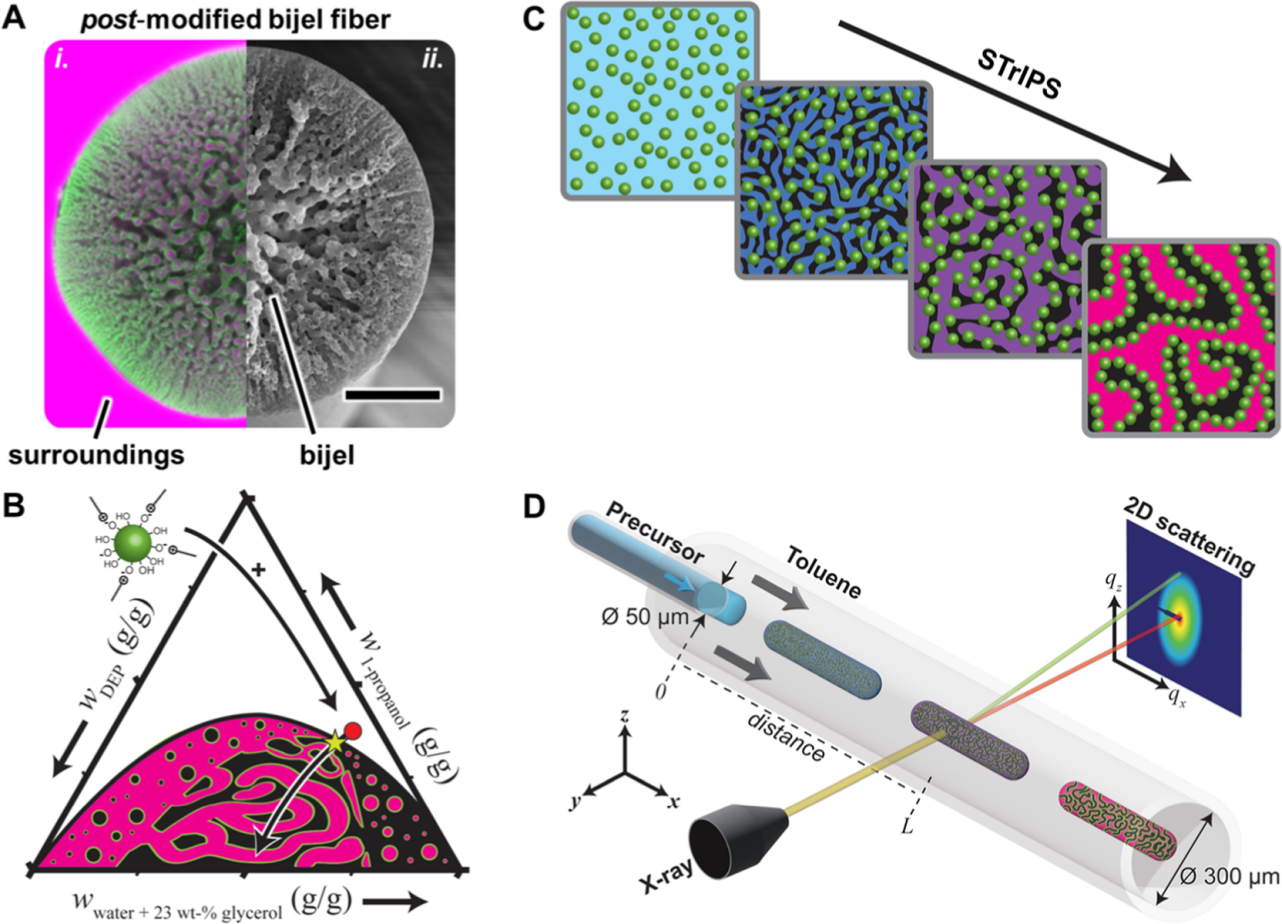
Bijel formation. (A) Cross-section of bijel fiber imaged by (i) confocal- and (ii) scanning electron microscopy. Magenta is oil, green is particles and black is aqueous phase. Scale bar = 20 μm. (B) Phase diagram of diethyl phthalate (DEP), water, glycerol and 1-propanol as weight fraction *w*_*i*_ with binodal curve, critical point (yellow star) and precursor liquid composition with CTAB-modified Ludox TMA particles (red dot). Illustration below the binodal represents the regions of spinodal decomposition and nucleation. (C) Schematic bijel formation via spinodal decomposition and kinetic arrest of liquid domains. (D) In situ microfluidic extrusion device for in situ measurements at a beamline.

### 2.7 X-Ray Scattering Experiments and Data Analysis

Static and time-resolved SAXS/USAXS measurements were performed at the ID02 time-resolved ultrasmall-angle X-ray scattering beamline at the European Synchrotron Research Facility (ESRF). The beam had an energy of 12.4 keV, corresponding to a wavelength of 0.1 nm and was 40 μm · 40 μm in size (fwhm). An Eiger2 4 M detector was used for both SAXS and USAXS measurements by varying the sample–detector distance between 3 m (SAXS) and 31 m (USAXS). These distances cover a scattering vector *q* between 1.73 × 10^–3^ to 2.5 nm^–1^ ( $q=\frac{4\pi }{\lambda }\sin\left(\frac{\theta }{2}\right)$ with θ as scattering angle and λ as wavelength).

Static measurements were conducted to characterize the structure of silica Ludox TMA nanoparticles. Quartz capillaries (1.5 mm diameter, wall thickness 10.0 μm) were loaded by aqueous Ludox TMA dispersions at pH 1.8 with weight fractions ranging between 1 × 10^–3^ and 0.50 g/g. The temperature in the experimental cabin was set to 23.5 °C. Ten frames with an exposure time of 0.10 s per frame were collected in both SAXS and USAXS.

Real-time scattering measurements on the extrusion of bijel fragment were performed by positioning the microfluidic device horizontally on a movable stage in the beamline. Different positions from the orifice onward were irradiated with step intervals between 25 μm and 5 mm. The position of the orifice was determined by exposure of the device at various positions until no beam refraction by the inner capillary was measured. Ten frames with an exposure time of 0.10 s per frame were collected in SAXS and 30 frames were collected for USAXS.

Recorded two-dimensional (2D) scattering patterns are normalized to an absolute intensity scale (see Supporting Information S6). One-dimensional (1D) scattering patterns *I*(*q*) were obtained by averaging 2D patterns over 360° for all SAXS measurements and for static measurements-only in USAXS according to standard procedures.^40^ 2D time-resolved USAXS measurements were averaged over azimuthal angles between −20° and +20° (see Supporting Information S5). 1D patterns were averaged using all frames measured per measurement and corrected for background scattering using SAXSutilities2 software as described in Supporting Information sections S4 and S5.^41^ Plotting and further data analysis has been done using OriginPro software.

Unless stated otherwise, all 1D scattering profiles and 2D scattering patterns shown throughout this paper are background corrected.

## 3 Results and Discussion

In this study, we focus on the formation of bijels with an interwoven submicron network interior. Figure 2A shows a cross-section of a bijel fiber imaged by confocal laser scanning microscopy (CLSM) and scanning electron microscopy (SEM). CLSM identifies the interwoven oil- (labeled magenta) and aqueous networks (labeled black) stabilized by a rigid particle layer (labeled green). SEM shows the range of pore sizes between 200 nm near the outer surface to several microns near the center. This pore size gradient evolves due to the formation mechanism of bijels as reported previously.^38,39^ Recent work showed that these bijels can be fabricated as fibers from a critical precursor mixture via solvent transfer induced phase separation (STrIPS).^38,39,42^ This precursor mixture contains oil (diethyl phthalate), aqueous (water and glycerol), solvent (1-propanol), nanoparticles (silica Ludox TMA, 29 nm) and surfactant (cetyltrimethylammonium bromide, CTAB) (see Figure 2B). During STrIPS, the precursor mixture is injected into toluene. 1-propanol diffuses from the precursor into toluene and triggers phase separation. Consequently, CTAB-modified silica nanoparticles self-assemble on the liquid–liquid interface and arrest the bicontinuous structure as schematically depicted in Figure 2C.

To study nanoparticle self-assembly during phase separation, in situ SAXS and USAXS experiments are performed at the ID02 beamline at the European Synchrotron Radiation Facility (ESRF), Grenoble, France (see Methods Section 2.7).^36^ A microfluidic device consisting of two coaxially aligned glass capillaries is horizontally positioned at the beamline, as illustrated in Figure 2D (see Method Section 2.3 and 2.4). Bijels are continuously extruded as pinched-off fragments by injecting a precursor mixture via an inner capillary into a flow of toluene in an outer capillary (see Method Section 2.5 and S2).^43^ While traversing through toluene, the fragments undergo STrIPS and form phase separated bijel structures. Measuring the scattering of fragments at various positions along the capillary enables in situ tracking of the transition from a homogeneous precursor mixture into phase separated structures. The positions are converted into time scales using the estimated velocity of the fragments through the capillary based on the flow rates of the precursor and toluene (see Supporting Information S2 and S3). These measurements provide time-resolved insights into phase separation and self-assembly.

In the following, we first explore the self-assembly kinetics on the nanoparticle level during phase separation as acquired by SAXS. Thereafter, we extend the discussion on the structural evolution to larger scales like bicontinuous network formation as probed by USAXS. Unless stated otherwise, the scattering intensity profiles presented in this paper have been normalized and background-corrected according to standard procedures (see Supporting Information S4 and S5).^36^

### 3.1 Nanoparticle Assembly during Phase Separation

We first study the X-ray scattering on the nanoparticle level when fragments undergo phase separation. Figure 3A shows the scattering intensity profiles *I*(*q*,*t*_*i*_) against the scattering vector *q*, recorded in situ during the formation of a bijel fragment for time *t*_*i*_ from 0 to 611 ms (see Supporting Information S6). Here, the measured *q*-values range from a maximum of 1.0 nm^–1^ to a minimum of 0.025 nm^–1^, corresponding to spatial scales around, respectively, 6 and 250 nm. In this *q*-range, the patterns are dominated by the scattering of silica nanoparticles (see Supporting Information S4 and S11).^44^ For *q* > 0.30 nm^–1^, *I*(*q*,*t*_*i*_) originates from the scattering of individual silica particles with fringes characteristic for spherical particles.^45^ Thus, to observe the particle organization and to reveal their spatial correlations during phase separation, one has to focus on the region below *q* = 0.30 nm^–1^.

**Figure 3:**
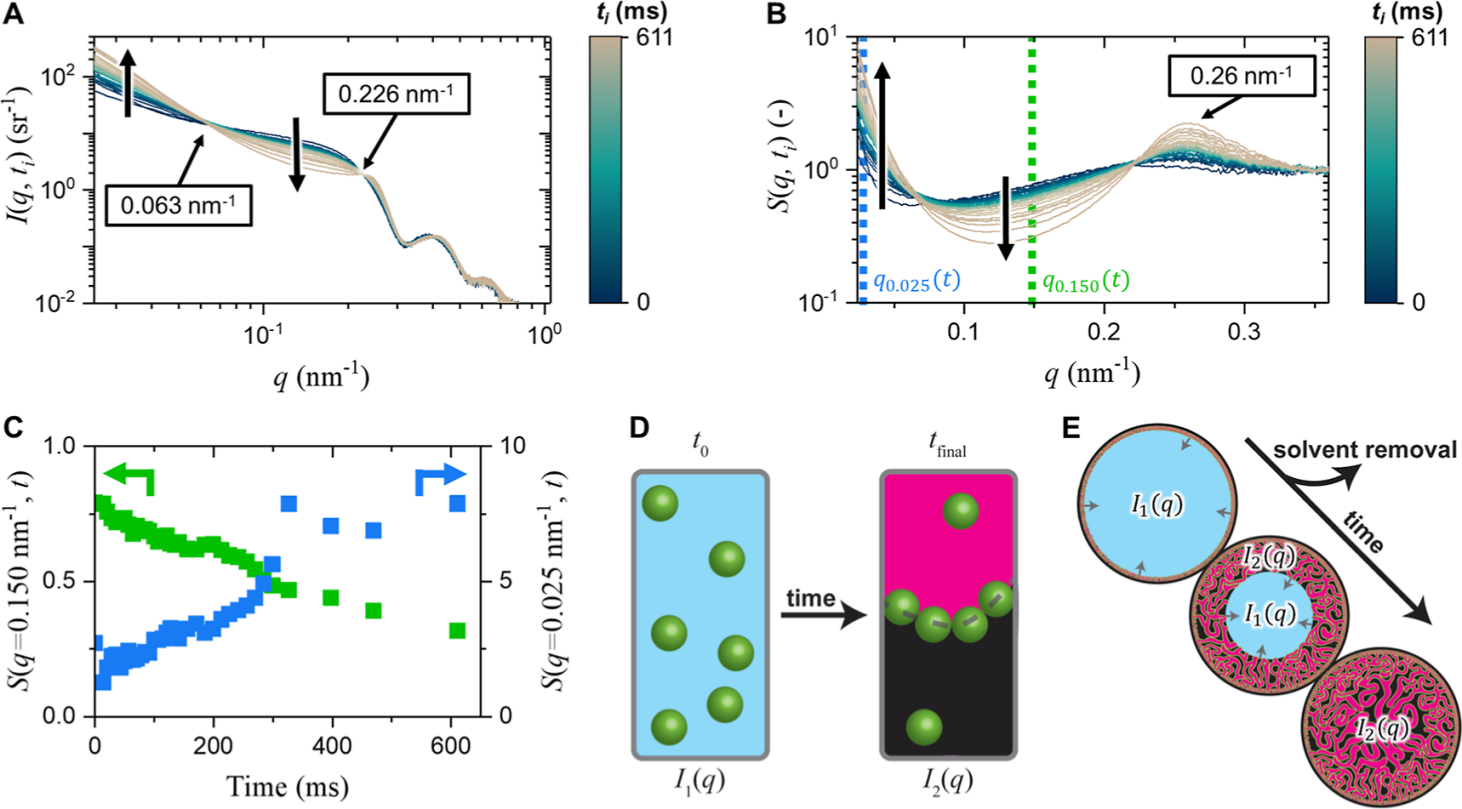
Nanoparticle assembly during phase separation acquired by in situ SAXS. (A) Normalized scattered intensity profiles on a double-log scale during bijel formation over time. (B) Normalized time-resolved structure factors on a log–linear scale during bijel formation. (C) Time-resolved structure factor at (green symbols, left axis) *q* = 0.150 nm^–1^, nearest neighbor interactions, and (blue symbols, right axis) *q* = 0.025 nm^–1^, larger structural contributions. (D) Proposed two-state system on particle assembly from randomly dispersed particles with scattering intensity *I*_1_(*q*) with free nanoparticle volume fraction ϕ into an ordered layer of adsorbed particles at the oil–water interface with scattering intensity *I*_2_(*q*). (E) Schematic representation of radially inward formation of bijel fragment during STrIPS and the contribution on the superposition of the scattering of *I*_1_(*q*) and *I*_2_(*q*).

In the region for *q* < 0.30 nm^–1^, two iso-scattering points in *I*(*q*,*t*_*i*_) can be observed at *q*_1_ = 0.063 and *q*_2_ = 0.226 nm^–1^. In between these iso-scattering points, *I*(*q*,*t*_*i*_) decreases over time. Simultaneously, the region below *q*_1_ = 0.063 nm^–1^ shows a strong upturn in intensity. These variations in *I*(*q*,*t*_*i*_) suggest that the positional correlation between nanoparticles changes over time, as discussed next.

We point out that *I*(*q*,*t*_*i*_) depends on structural interactions between the particles. The scattering intensity *I*(*q*) can be factorized into the product of a form factor *P*(*q*) and structure factor *S*(*q*) via *I*(*q*)∝*P*(*q*)*S*(*q*). Here, *P*(*q*) describes the scattering of individual silica nanoparticles (Supporting Information S7). *S*(*q*) depends on the positional correlations between particles and can be extracted by dividing *I*(*q*) by *P*(*q*), followed by normalizing the high *q* limit to 1 (see Supporting Information S8).

Figure 3B shows the structure factor *S*(*q*,*t*_*i*_) calculated from the scattering intensity profiles *I*(*q*,*t*_*i*_) for *t*_*i*_ between 0 and 611 ms (see Supporting Information S8). Initially, *S*(*q*,*t*_*i*_) exhibits only slight variations as a function of *q* as expected for a dilute suspension. Over time, three main features in *S*(*q*,*t*_*i*_) can be observed: (I) a maximum develops at *q*_max_ = 0.26 nm^–1^, (II) *S*(*q*,*t*_*i*_) reduces between the iso-scattering points, and (III) *S*(*q*,*t*_*i*_) sharply increases for the region below iso-scattering point *q*_1_.

The first two changes mentioned provide structural insights into the self-assembly of the nanoparticles over time. The correlation peak *S*(*q*_max_ = 0.26 nm^–1^,*t*_*i*_) indicates the typical interparticle correlation distance (see Supporting Information S8).^46^ In real space, this peak corresponds to 24 nm, which is similar to the correlation distance in a hexagonal monolayer of particles that equals $\sqrt{3/4}$ times the particle diameter (29 nm). Besides, the reduction in *S*(*q*,*t*_*i*_) between the iso-scattering points indicates reduced density fluctuations on the scale of neighboring particles, which is characteristic for the formation of densely packed structures. Together, these two effects suggest that nanoparticles self-assemble from a homogeneous dispersion into closely packed structures during phase separation. The third change in *S*(*q*,*t*_*i*_) i.e. the sharp increase for *q* < 0.063 nm^–1^, indicates that the closely packed particles are organized into larger structures during the self-assembly. This intensity upturn can be used to identify the onsets and kinetics of particle self-assembly and formation of larger structures.

To this end, *S*(*q*,*t*_*i*_) has been plotted against time at arbitrary *q* values of 0.150 nm^–1^ and 0.025 nm^–1^, corresponding to, respectively, the self-assembly of individual nanoparticles and the formation of larger structures, see Figure 3C. This plot shows that both *S*(*q* = 0.150 nm^–1^,*t*) and *S*(*q* = 0.025 nm^–1^,*t*) profiles change monotonically from the beginning of the extrusion onward. However, whereas *S*(*q* = 0.150 nm^–1^,*t*) continuously decreases for the full 600 ms measured, *S*(*q* = 0.025 nm^–1^,*t*) reaches a plateau from 350 ms and remains nearly constant at longer times. This difference between the time dependences of the structure factor is not clear yet and needs further investigation.

The presence of the iso-scattering points in the scattering data suggests that particles are present in two competing states during phase separation. In this case, the scattering of one state, *I*_1_(*q*), would originate from single nanoparticles dispersed in a liquid medium with a flat structure factor. During phase separation, however, the nanoparticles start to adsorb at the interfaces with a condensed structure with a different *I*_2_(*q*) profile as illustrated in Figure 3D. Since the condensed phase grows at the cost of the dispersed phase, their contributions can be described by a single parameter ϕ­(*t*), which is the time-dependent fraction of freely dispersed particles. As discussed in more detail in Supporting Information S9, the main features of the measured *I*(*q*,*t*) can be simulated using *I*(*q*,*t*) = ϕ­(*t*)*I*_1_(*q*)+(1-ϕ­(*t*))*I*_2_(*q*). The time dependence of the ϕ­(*t*) parameter can describe the radial inward propagation of the phase separation front from the outer surface of the fiber fragment toward its center as shown in Figure 3E.

In summary, the SAXS data indicate that the transition from a single phase with uniformly distributed silica nanoparticles to their dense assemblies at the interfaces, takes at least 600 ms. In addition, the strong intensity upturn at smaller *q* indicates the formation of larger structures, which will be discussed in more detail below with the USAXS data.

### 3.2 Bicontinuous Network Channel Formation

In the following, we will extend the discussion to ultrasmall X-ray scattering (USAXS) to analyze scattering patterns for *q*-ranges corresponding to the micron scale (see Supporting Information S5). Figure 4A displays the evolution of the 2D scattering patterns for early stages of the STrIPS process up to 67 ms. Initially, we see strong isotropic scattering in the first few milliseconds after the extrusion. Surprisingly, most of this signal disappears for about ∼50 ms. After this time, strong scattering starts to develop at *q*-values corresponding to submicron scales. The intensity of this scattering pattern grows in time and shifts simultaneously toward lower *q*-values. Remarkably, this scattering appears strongly anisotropic and is mostly observed along the fiber direction.

**Figure 4:**
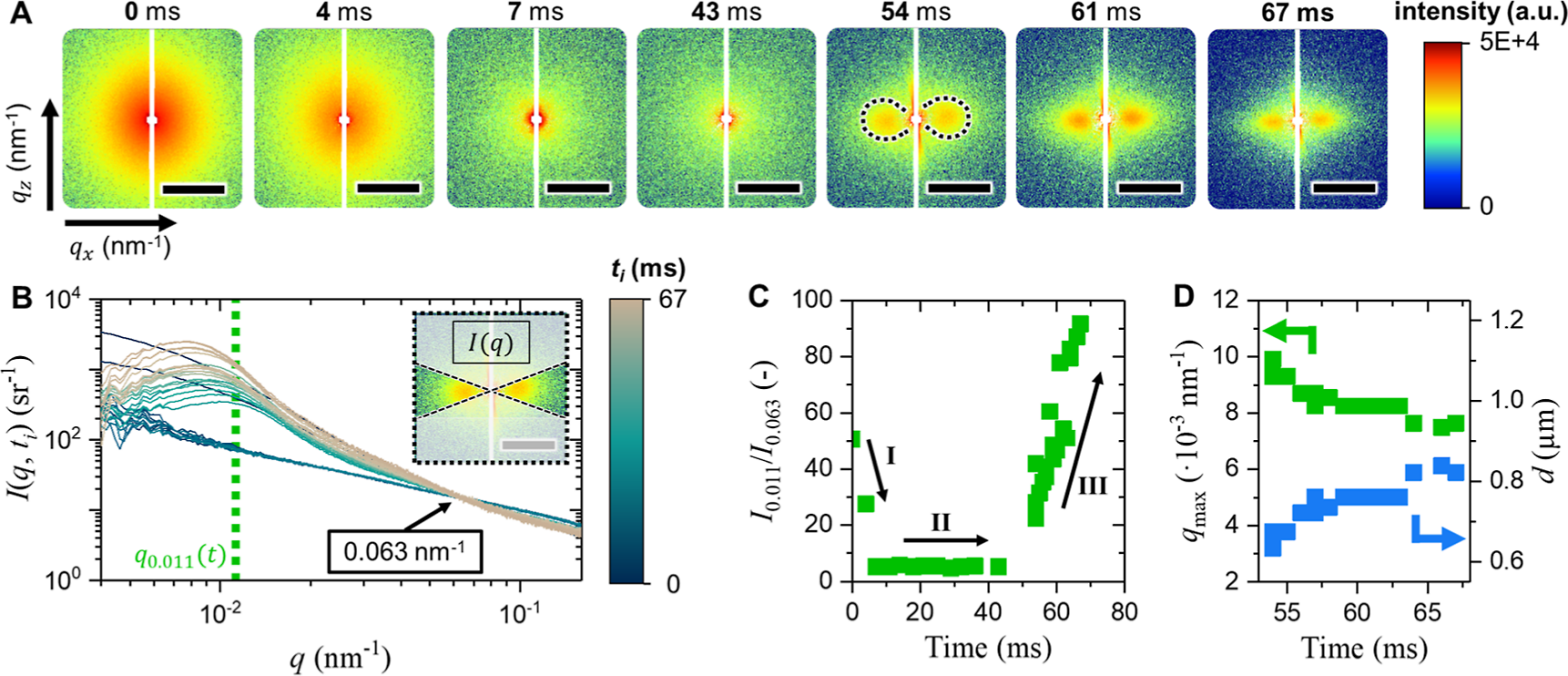
In situ USAXS analysis on bijel formation. (A) 2D scattering patterns of bijel fragments after various time steps. The dashed lines in the frame of 54 ms indicate the circumference of the lobes formed. (B) Normalized scattering 1D intensity plots after averaging the bright region of the 2D scattering pattern shown in the inset. Normalization has been done at the iso-scattering point at *q*_1_ = 0.063 nm^–1^ as found by SAXS in Figure 3A. (C) Time-dependence of the normalized intensity at *q* = 0.011 nm^–1^ with phases I, II and III indicated. (D) Time-dependence of position of the broad correlation maximum *q*_max_ and corresponding characteristic interwall distance against STrIPS time determined from the structure factor (see Supporting Information S8).

To better understand the evolution of these 2D scattering patterns, the 1D intensity scattering profiles around the *q*_*x*_ axis are analyzed. Figure 4B shows *I*(*q*,*t*_*i*_) profiles averaged over an azimuthal angular range of ±20° as shown in the inset of Figure 4B (see Supporting Information S5). Similar to SAXS data, the amount of material within the irradiated area fluctuates because of extrusion instabilities, undulation of the fiber, and so on. To remove these intensity fluctuations, the *I*(*q*,*t*_*i*_) profiles are normalized to the same intensity at the iso-scattering point at *q*_1_ = 0.063 nm^–1^ as earlier found by SAXS. For *q* < 0.063 nm^–1^, a strong scattering intensity can be seen in the first few milliseconds and after longer times in agreement with the 2D scattering patterns. However, the strong scattering signals in the first few ms are different than after 50 ms. The former is isotropic and exhibits no maximum. The latter is anisotropic and shows a broad maximum as a function of *q*.

To quantify the evolution of the scattering profiles over time, Figure 4C plots *I*(*q*,*t*_*i*_) against time at an arbitrarily selected *q* = 0.011 nm^–1^ normalized by the intensity at *q*_1_. This plot shows that three phases in *I*(*q* = 0.011 nm^–1^,*t*) can be observed: (I) a rapid decrease within 7 ms, (II) a constant intensity until 50 ms, and (III) a strong increase from 50 ms onward.

In addition, Figure 4D plots the position of the broad maximum peak in phase III, *q*_max_, against time, and the corresponding distance, *d* = 2π/*q*_max_, in direct space. This plot shows that *q*_max_ lowers from 10 × 10^–3^ to less than 8 × 10^–3^ nm^–1^, corresponding to an increase from 0.6 μm to over 0.8 μm in real space within 67 ms. No longer times have been measured for this extrusion due to practical constraints.

What does the evolution of the scattering profiles reveal about the synthesis of bijel fragments formed via STrIPS? The three distinct phases in *I*(*q* = 0.011 nm^–1^,*t*) suggest that the formation occurs via three consecutive steps. To further elaborate on this question, we point out that the scattering intensity measured in USAXS originates from both nanoparticles and phase separated liquids (see Supporting Information S11). We also note that the Teubner–Strey model that is usually used to describe scattering by phase-separating liquids, cannot be directly applied to our data due to the presence of nanoparticles with a high scattering contrast.^47^

We begin the discussion of the USAXS starting from phase I, which corresponds to the injection of the precursor mixture into the stream of toluene. The strong scattering observed here is not present in a similar USAXS measurement performed under static conditions (see Supporting Information S10). The strong scattering in phase I likely arises from the flow patterns generated in the precursor mixture by toluene. Just before the orifice, the precursor mixture has a parabolic velocity profile inside the inner capillary. After extrusion, the outer part of the precursor mixture has to quickly accelerate to catch up with the faster toluene flow (see Supporting Information S3). In fact, the initial scattering profiles can be described by the scattering function of highly elongated parallelepipeds. These may well correspond to the stretched lamellae forms expected at the onset of mixing.^48^ As the mixing progresses, these lamellae become thinner, folded and then fade away as the concentration differences diminish.^49^ This is indicated by the rapid drop in intensity, which may have also partly caused by the movement of these patterns out of the tiny scattering volume.

Proceeding to phase II, *I*(*q*,*t*) remains low, indicating that no larger structures are formed in the precursor. This suggests that the precursor did not undergo yet phase separation. We conclude that there is a particular induction phase before the concentration of 1-propanol is sufficiently lowered to induce phase separation. In agreement with the phase diagram in Figure 2B, the precursor mixture contains a small excess of 1-propanol required to form a stable miscible mixture.

Finally, the strong increase in *I*(*q*,*t*) in phase III indicates that larger structures are being formed. The correlation peak present in the scattering intensity indicates that a bicontinuous structure is formed via spinodal decomposition. The strongly anisotropic shape of the correlation peak suggests that the formed structures are influenced by the presence of a shear force,^50–53^ and the inward progression of the phase separation leading to the radial orientation of the pores in a bijel fiber as shown in Figure 2A.

Before concluding, we compare the kinetic phenomena involved in bijel formation via STrIPS of the precursor mixture used in this study. Previous work modeled the diffusion of 1-propanol out of the precursor mixture and found that the radially inward-directed phase separation begins from 20 ms after extrusion.^38^ In the present study, USAXS indicated an induction phase of 50 ms before spinodal decomposition occurs, similar to the time scale predicted by simulations. In contrast, SAXS revealed that some nanoparticles already self-assemble locally within the induction period of 50 ms.

We attribute this earlier particle self-assembly to the local phase separation and self-assembly in the vicinity of the outer-surface of the precursor mixture which is in direct contact with toluene, forming small structures on the submicron scale. The formation of the larger, (sub)­micron sized structures occurs after the induction phase of 50 ms. At this time, the radial inward propagation of the spinodal decomposition of the precursor mixture starts, resulting in the consequent network formation as depicted in Figure 2C.

## 4 Conclusion

To summarize, combined time-resolved SAXS and USAXS measurements allow real-time monitoring of the complex formation of bicontinuous emulsion gels, a process previously experimentally inaccessible. Synchrotron SAXS/USAXS enables precise characterization of liquid phase separation and colloidal self-assembly on a millisecond time scale. SAXS measurements capture the transition from individually dispersed particles to condensed self-assembled structures, while USAXS elucidates the formation of larger structures composed of both liquids and particles. For the first time, in situ experimental evidence confirmed that bijels synthesized from a liquid precursor mixture undergoing solvent transfer induced phase separation (STrIPS) are formed via spinodal decomposition. In addition, we have identified that the formation of such bijels occurs via three consecutive steps: kinetic phenomena related to fluid dynamics within the first 7 ms, local nanoparticle self-assembly between 7 and 50 ms, and the formation of the (sub)­micron structures with the inward propagation of the liquid–liquid phase separation from 50 to 600 ms. While the exact mechanisms of these steps remain unclear, it offers opportunities for further investigation. The findings already presented in this work provide new insights into the kinetically controlled formation of bicontinuous emulsion gels. It opens up new analysis methods to characterize the synthesis mechanisms of various complex nanostructures. Eventually, this research can improve the understanding of complex assembly phenomena and enhance the potential of future applications.

## Supplementary Material





## Notes

Fiber extrusion versus fragment extrusion; velocity profile through capillary; SAXS background subtraction and liquid scattering; USAXS background subtraction and data reduction; normalization of measured *I*(*q*,*t*_*i*_); SAXS Form factor of aqueous Ludox TMA nanoparticles; calculating SAXS structure factor during bijel extrusion; simulation of *I*(*q*,*t*_*i*_) and *S*(*q*,*t*_*i*_) within two-phase model; scattering intensity of flowing and static precursor; calculation of scattering length density (SLD) and their effect on the contrast in SAXS and USAXS (PDF)
Real-time extrusion of bijel fiber and fragments in microfluidic extrusion device. (Video S1, AVI) (MP4)

## §

TNO Environmental Modeling, Sensing and Analysis, Princetonlaan 6, 3584CB Utrecht, The Netherlands
