# Supplementary material for: Insights into Formation of Bicontinuous Emulsion Gels via In Situ (Ultra-)Small Angle X‑Ray Scattering
Source: J Phys Chem B. 2025 Jun 12;129(25):6419–27. doi: 10.1021/acs.jpcb.5c02375 (PMC12207573; doi:10.1021/acs.jpcb.5c02375)
Supplement: Supplementary file 1 [file jp5c02375_si_001.pdf]

Supporting Information

Insights into Formation of Bicontinuous Emulsion Gels via *in-situ* (Ultra-)Small Angle X-ray Scattering

Meyer T. Alting[1], Dominique M.E. Thies-Weesie[1], Alexander M. van Silfhout[1,2], Mariska de Ruiter[1], Theyencheri Narayanan[3], Martin F. Haase[1], Andrei V. Petukhov[1*]*

[1] Van 't Hoff Laboratory for Physical and Colloid Chemistry, Department of Chemistry, Debye Institute for Nanomaterials Science, Utrecht University, Padualaan 8, 3584CH, Utrecht, The Netherlands

[2] *Current affiliation*: TNO Environmental Modelling, Sensing and Analysis, Princetonlaan 6, 3584CB Utrecht, The Netherlands

[3] ESRF – The European Synchrotron, 71 Avenue des Martyrs, 38043, Grenoble, France

* Correspondence: a.petoukhov@uu.nl and m.f.haase@uu.nl

Supporting Information 1 Supplementary Video Captions 2
Supporting Information 2 Fiber extrusion versus Fragment extrusion 2
Supporting Information 3 Velocity profile through capillary 3
Supporting Information 4 SAXS background subtraction and liquid scattering 5
Supporting Information 5 USAXS background subtraction and data reduction 6
Supporting Information 6 Normalization of measured $\boldsymbol{I(q,ti)}$ 7
Supporting Information 7 SAXS Form factor of aqueous Ludox TMA nanoparticles 7
Supporting Information 8 Calculating SAXS structure factor during bijel extrusion 8
Supporting Information 9 Simulation of $\boldsymbol{I(q,ti)}$ and $\boldsymbol{S(q,ti)}$ within two-phase model 8
Supporting Information 10 Scattering intensity of flowing and static precursor 11
Supporting Information 11 Calculation of Scattering Length Densities (SLD) and their effect on the contrast in SAXS and USAXS 12

Supporting Information 1 Supplementary Video Captions

Supplementary Video: Video of extrusion of fiber and pinched-off fragments in a microfluidic device as described in detail in SI section 2.

Supporting Information 2 Fiber extrusion versus Fragment extrusion

For analytical purposes and applications, bijels are continuously extruded as fibers (**Figure S2)**. Extrusion in microfluidic devices occurred inhomogeneously as fibers undulated, stuck to the glass and formed large accumulates in the capillaries (see SI video). These factors affect the time-resolved interpretation of bijel formation using the positional measurements. Alternatively, extruding fragments showed homogeneous movement throughout the capillary. As the length of the fiber was significantly larger than the diameter, we assumed that the phase separation kinetics are similar as in a fiber.

Fiber extrusions were typically performed using a precursor flowrate of 0.80 mL/h and 5.00 mL/h for toluene. Fragments are extruded using flowrates of 0.40 and 17.50 mL/h for, respectively, precursor and toluene. These fragments were extruded as a rate of ~30 s^{-1} with an average spacing of 3 – 5 mm between the fragments.

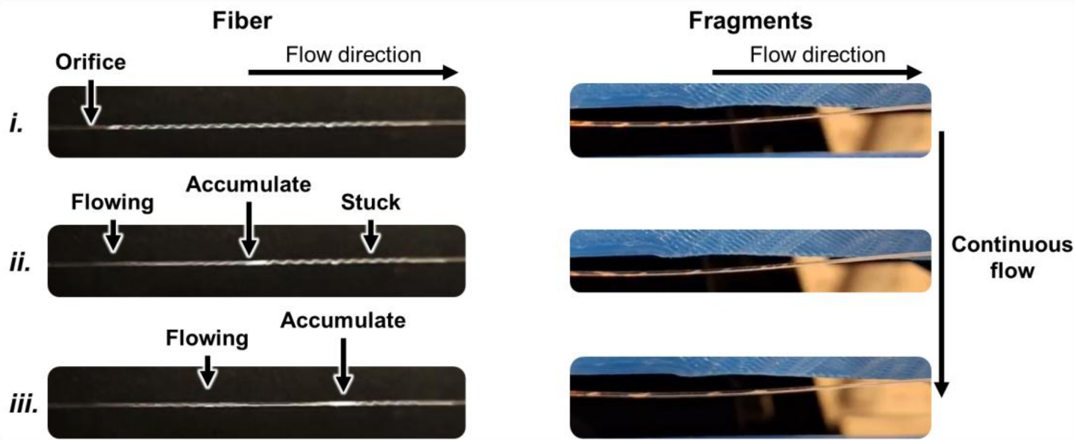

Figure S2: Time recordings of the extrusion of (left) fibers, and (right) fragments of bijel.

Supporting Information 3 Velocity profile through capillary

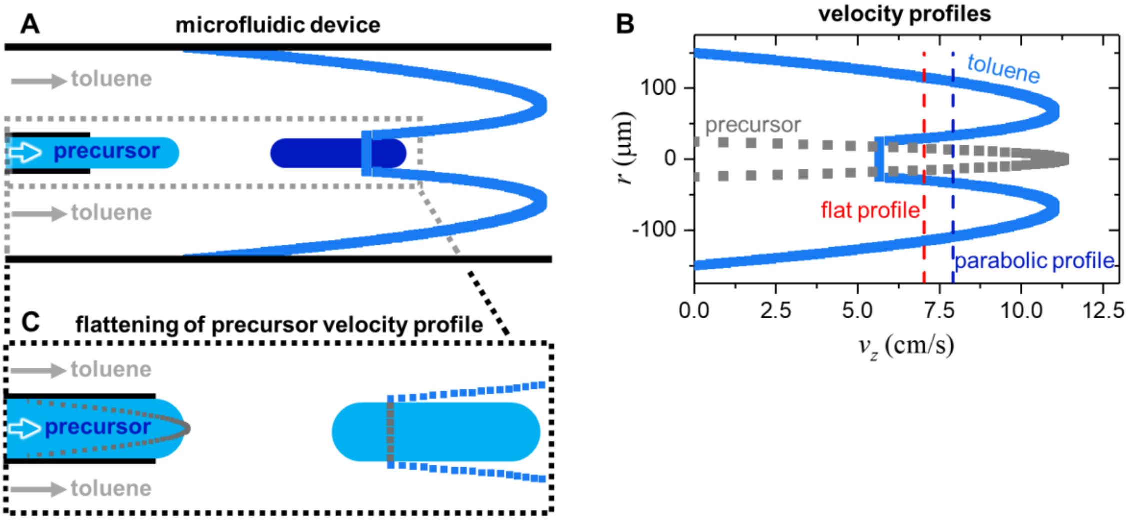

Figure S3: Velocity profiles in microfluidic device during fragment extrusion. **A)** Illustration of velocity profile in microfluidic device. **B)** Plot of velocity profiles against radial position r for (grey squares) precursor in 50 µm diameter inner capillary with $Q_{\text{precursor}}$ = 0.40 mL/hr, (blue squares) toluene in 300 µm diameter outer capillary with Q_{toluene} = 17.50 mL/hr, (blue dashed line) averaged parabolic velocity and (red dashed line) flat profile. **C)** Proposed flattening of precursor velocity profile before and after injection.

The velocity of an extruded bijel fragment in a microfluidic device can be estimated assuming either a Laminar flow or constant profile in the outer capillary. The average velocities between both methods hardly differ as discussed next.

The Laminar velocity profile $v_z(r)$ can be calculated by treating the fragments as solid-like materials, as illustrated in **Figure S3A**. Using cylindrical coordinates and assuming no slip boundary conditions on the capillary walls ($v_z(r = R_2) = 0$) and fragment surface ($v_z(r = R_1 = U)$, $v_z(r)$ is calculated as:[1]

$$v_z(r) = -\frac{\mathrm{d}P}{\mathrm{d}z}\left(\frac{R_2^2 - r^2}{4\mu}\right) + \left(U + \frac{\mathrm{d}P}{\mathrm{d}z}\left(\frac{R_2^2 - R_1^2}{4\mu}\right)\right)\frac{\ln\left(\frac{R_2}{r}\right)}{\ln\left(\frac{R_2}{R_1}\right)}$$

with

$$\frac{\mathrm{d}P}{\mathrm{d}z} = \frac{2\mu\ln\left(\frac{R_2}{R_1}\right)}{\pi J}\left(Q_{\text{tol}} + \frac{2\pi U}{\ln\left(\frac{R_1}{R_2}\right)}\left[\frac{R_1^2}{2}\ln\left(\frac{R_1^2}{2}ln\left(\frac{R_1}{R_2}\right)\right) + \frac{R_2^2}{4} - \frac{R_1^2}{4}\right]\right)$$

and

$$J = \left(\frac{R_1^4}{4} - \frac{R_2^4}{4}\right)\ln\left(\frac{R_2}{R_1}\right) + \left(\frac{R_2^2}{2} - \frac{R_1^2}{2}\right)^2$$

and

$$U = \frac{Q_{\text{prec}}}{\pi R_1^2}$$

where R_1 is the fiber's radius, R_2 the outer capillary's radius, μ the viscosity of toluene, U the translational velocity of the fragment, Q_{prec} and Q_{tol} the flow rate of, respectively, precursor and toluene.

As the fragment slightly undulates in the capillary, the average fiber velocity has been calculated via

$$v_{z,average} = \frac{1}{2R_2}\int_{-R_2}^{R_2} v_z(r)\mathrm{d}r$$

Using the experimental conditions of R_1 = 50 µm, R_2 = 150 µm, Q_{toluene} = 17.50 mL/hr, $Q_{\text{precursor}}$ = 0.40 mL/hr and μ_{toluene} = 0.59 mPa·s (20 °C) gives $v_{z,average}$ = 7.9 cm/s as indicated in **Figure S3B**.

The undulation of the fragment disrupts the velocity profile, affecting the actual value for $v_{z,average}$. Alternatively, a flat velocity profile has been calculated via:

$$v_{fragment} = \frac{Q_{\text{precursor}} + Q_{\text{toluene}}}{\pi R_2^2}$$

Using the same experimental conditions gives v_{fiber} = 7.0 cm/s as indicated in **Figure S3B**. This shows that both methods gives similar average velocities. Using $v_{fragment}$ allows to compare phase separation kinetics across different experimental conditions while the actual time scale may slightly vary. In the main text, the time scales during extrusion have been estimated from the flat velocity profile.

The velocity profile of the precursor inside the 50 µm inner capillary can be calculated assuming $v_z(r = R) = 0$ i.e. no slip boundary condition at capillary wall via:[1]

$$v_z(r) = \frac{2}{\pi R^2} Q\left(1 - \left(\frac{r}{R}\right)^2\right)$$

The faster flow of toluene exerts a shear force on the fragment's outer surface upon extrusion, increasing the fragment's velocity. As a result, the velocity profile near the outer surface increases from 0 to U (as described before) during extrusion. This may flatten the velocity profile in the fragment (**Figure S3C**).

Supporting Information 4 SAXS background subtraction and liquid scattering

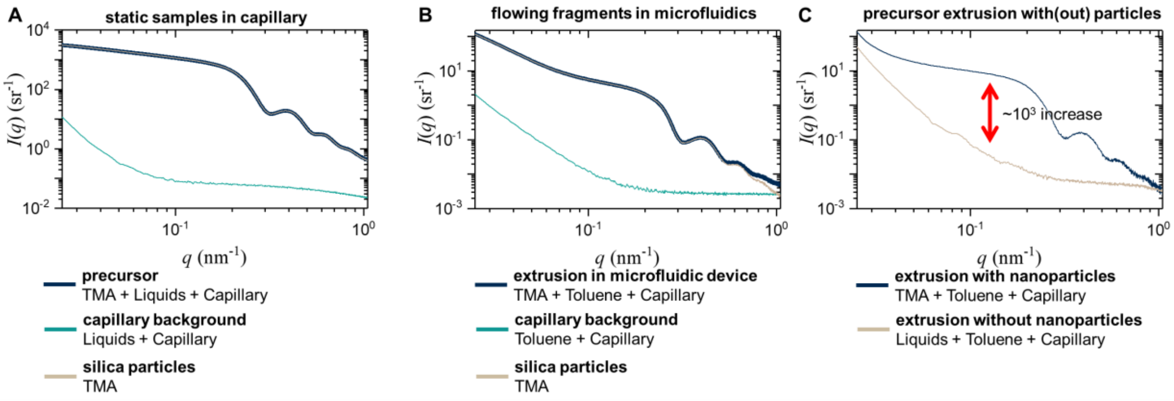

Figure S4: Background subtraction procedure for $I(q)$ for **A)** static samples in capillaries, and **B)** flowing samples in microfluidic devices. **C)** *In-situ* extrusion of precursor mixtures with and without nanoparticles at $t = 0$ ms of extrusion before background subtraction.

Data subtraction on static and flowing samples have different background references. In static capillary samples, $I(q)$ of a precursor mixture contains scattering of silica TMA particles, CTAB, liquid mixture and quartz glass walls (**Figure S4A**). The effect of CTAB has been ignored as preliminary measurements showed no significant scattering due to the surfactant (not shown). The scattering of a quartz capillary filled with the liquid components of the precursor is significantly weaker than the precursor including silica nanoparticles. Scattering of only silica TMA particles has been calculated by subtracting the scattering of the quartz capillary filled with the liquid components of the precursor. The subtracted scattering of silica particles overlaps with the signal of the precursor. This shows that the precursor's scattering is dominated by silica nanoparticles independent of liquids and quartz glass capillary.

Upon extrusion, the precursor's liquid composition changes due to the removal of 1-propanol. We assume the scattering of the bijel fragment originates solely from the nanoparticles due to weak scattering of the liquids. Therefore, the measured $I(q)$ during extrusion consists of the scattering of nanoparticles, toluene and the borosilicate glass capillary (**Figure S4B**). Subtraction of the scattering of a microfluidic capillary solely filled by toluene results in the scattering of the silica nanoparticles. The subtracted $I(q)$ mostly overlap with the unsubtracted scattering; limited deviations for $q > 0.7$ nm^{-1} are observed. This range has been neglected as it solely corresponds to the intraparticle structure i.e. no interparticle structural contributions.

The measured intensity during extrusions under SAXS conditions is dominated by the scattering of nanoparticles, as shown in **Figure S4C**. From $q < 0.03$ nm^{-1}, the scattering of the liquids approaches similar scattering intensities, revealing the similar scattering intensities of both phase-separated liquids and nanoparticles under USAXS conditions (more details in SI S11).

Supporting Information 5 USAXS background subtraction and data reduction

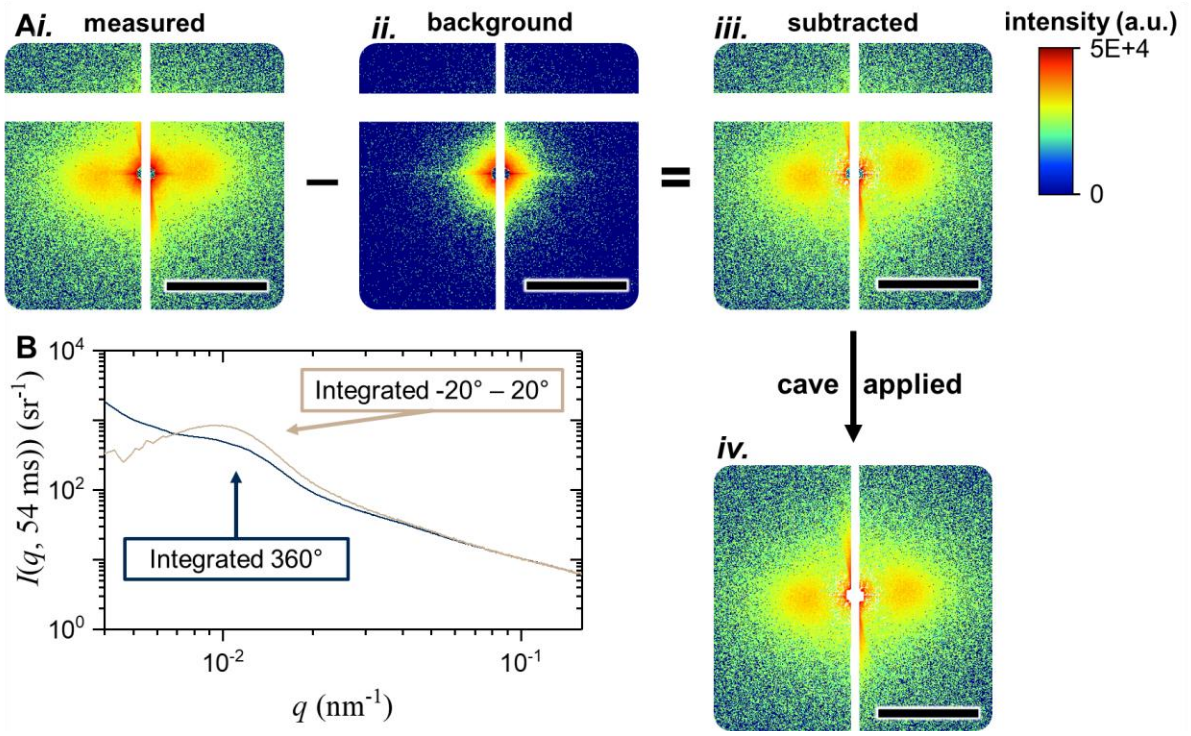

Figure S5: Background correction procedure of measured USAXS data from an extruded bijel fragment after 54 ms of extrusion. **A**) Different steps in background correction of 2D scattering patterns. Scale bar is 0.010 nm^{-1}. **B**) Comparison between 1D $I(q, 54\ \mathrm{ms})$ for integration of (blue) 360°, and (sand) azimuthal -20°-+20°.

The measured scattering profiles during extrusions have a large contribution of the background for $q < 10^{-2}$ nm^{-1}. A microfluidic device filled with toluene has been subtracted from the measured scattering using SAXSutilities2 / 2D tools (**Figure S5A**). For better representation of the 2D patterns, the horizontal shadow region between the individual detectors has been patched by mirroring (caving) the images along the horizontal and vertical planes (**Figure S5Aiv**).

Data reduction of the corrected data is done by integrating between azimuthal angles of -20° and 20° to characterize anisotropic features in the 2D patterns. **Figure S5B** shows reduced 1D $I(q, 54\ \mathrm{ms})$ profiles integrated over 360° or azimuthal angles between -20° and 20°. An obvious difference in $I(q, 54\ \mathrm{ms})$ can be seen for $q < 10^{-2}$ nm^{-1}: a shoulder in $I(q, 54\mathrm{ms})$ is present in the measured data, whereas a maximum is observed at $q = 10^{-2}$ nm^{-1} in the corrected data.

Supporting Information 6 Normalization of measured $\boldsymbol{I(q,t_i)}$

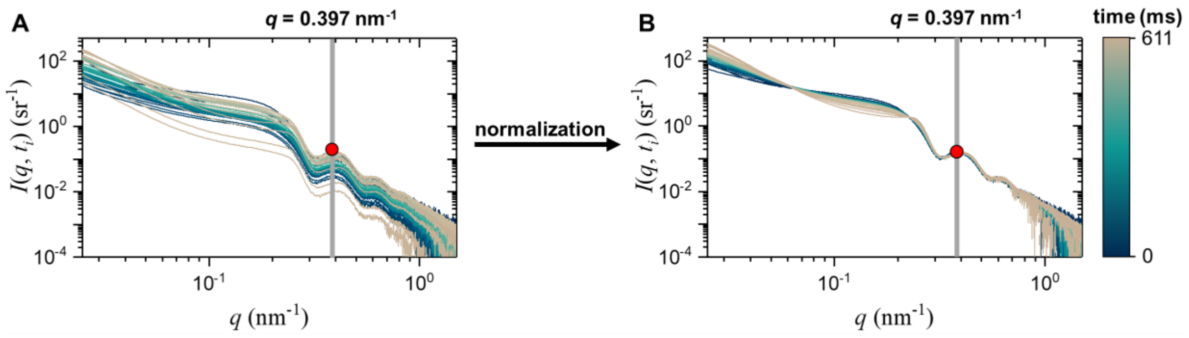

Figure S6: Normalization procedure of scattered intensity profiles in SAXS. **A)** $I(q,t_i)$ acquired during extrusion. **B)** Normalized $I(q,t_i)$ by using $I_i(q = 0.397\text{ nm}^{-1}) = I_0(0.397\text{ nm}^{-1})$.

The number of irradiated nanoparticles varies per measurement due to practical effects, resulting in an off-set change in $I(q,t_i)$ as shown in **Figure S6A**. To account for this variation, normalization has been done for the scattering of an individual nanoparticles by setting $I(q = 0.397\text{ nm}^{-1}, t_i)$ equal to $I(q = 0.397\text{ nm}^{-1}, 0\text{ ms})$ as shown in **Figure S1B**. The scattering for q > 0.30 nm^{-1} solely originates from silica nanoparticles and the scattering profiles after renormalization closely fall on top of each other. Thus, this renormalization allows to compare the different $I(q,t_i)$ profiles also at lower q-values.

Supporting Information 7 SAXS Form factor of aqueous Ludox TMA nanoparticles

Figure S7 shows the scattering profiles of aqueous Ludox TMA dispersions acidified to pH 1.8 at various weight fractions measured in sealed quartz capillaries (diameter 1.5 mm, wall thickness 10 μm). A background scattering of water at pH 1.8 has been subtracted from the measured scattering profiles. The form factor for Ludox TMA, $P_{TMA}(q)$, is set as $I(q)$ for w_i = 0.005 $g_{SiO2}/g_{dispersion}$. This is the lowest concentration with a proper signal-to-noise ratio and without structural development for q < 0.3 nm^{-1}. For dilute suspensions the intensity upturn at $q \to 0$ is lower than q^{-1} and presumably caused by the presence of small aggregated in the stock dispersion.

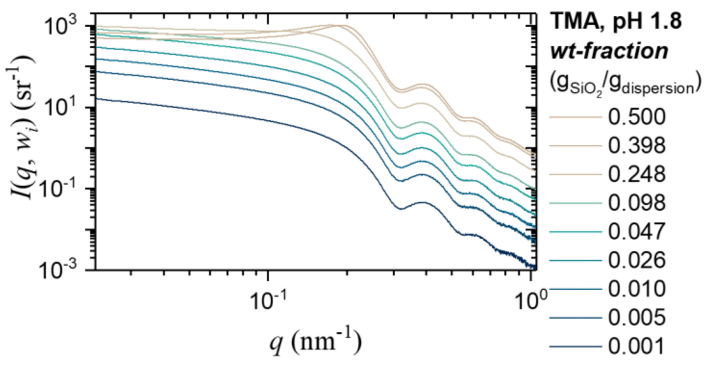

Figure S7: $I(q,w_i)$ of aqueous Ludox TMA dispersions at various wt-fractions.

Supporting Information 8 Calculating SAXS structure factor during bijel extrusion

The structure factor is extracted using the factorization of $S(q, t_i) = \frac{I(q,t_i)}{P(q)}$. **Figure S8A** shows the division of $I(q, t_i)$ of a bijel fragment after 171 ms of extrusion by the form factor $P(q)$ of silica TMA nanoparticles (SI S7) corresponding to scattering originating from individual particles without interactions.. As the number of silica particles within the beam is impossible to quantify, $S(q, t_i)$ profiles are normalized using $S(q = 0.35\ \text{nm}^{-1}, t_i) = 1$ (**Figure S8B**) as signal originates from particles.

$S(q, t_i)$ often contains a maximum correlation peak q_{max} located at $q > 0.2$. Assuming the Bragg relation is valid, the characteristic interparticle correlation distance (measured from core to core) is the reciprocal of the peak position (**Figure S8B**) given by $d = \frac{2\pi}{q_{\text{max}}}$. Assuming a 2D hexagonal monolayer, this correlation distance d equals 24 nm, which equals the particle diameter p_{particle} using $d = \sqrt{\frac{3}{4}} d_{\text{particle}}$, where d_{particle} equals 28 nm, which is similar to the particle diameter (29 nm).

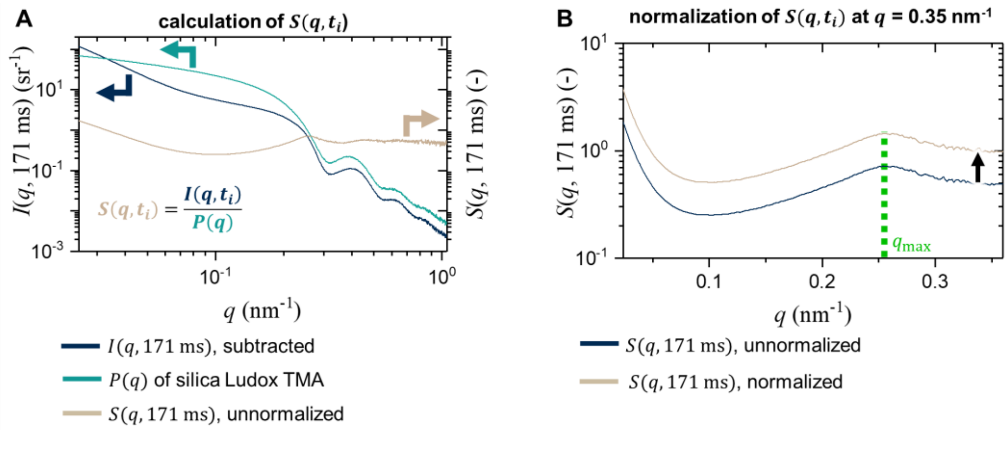

Figure S8: <u>Calculating $S(q, t_i)$ under SAXS conditions.</u> **A)** Conversion of $I(q, 171\ \text{ms})$ into $S(q, 171\text{ms})$ for bijel extrusion after 171 ms. **B)** Normalization of $S(q, 171\ \text{ms})$ using $S(q = 0.35\ \text{nm}^{-1}, t_i) = 1$ with indication of q_{max} (green dashed line).

Supporting Information 9 Simulation of $I(q, t_i)$ and $S(q, t_i)$ within two-phase model

Bijel formation is proposed to occur via a two-phase model system describing the irreversible attachment of nanoparticles to the liquid-liquid interface. As phase-separation occurs radially inward into the cylindrical fragment, the measured scattered intensity $I_{meas}(q, t)$, can be described as the superposition of the scattering intensity of free and attached nanoparticles via (**Figure 2E** in main text):

$$I_{meas}(q, t) = \phi(t) I_1(q) + \big(1 - \phi(t)\big) I_2(q)$$

where $I_1(q)$ is the scattering intensity of freely dispersed particles, $I_2(q)$ of attached nanoparticles and $\phi(t)$ the time-dependent volume fraction of free nanoparticles.

We describe $\phi(t)$ via $\phi(t) = A\exp(-at)$. Using boundary conditions $\phi(0) = \phi_0$ and $\phi(t_f) = \phi_f$, where ϕ_0 and ϕ_f are, respectively, the initial and final volume fraction of freely dispersed nanoparticles gives as expression $\phi(t) = \phi_0\left(\exp\frac{\ln\left(\frac{\phi_0}{\phi_f}\right)}{t_f}t\right)$. t_f has been determined from the experimental measurement when no significant change in $I(q)$ occurs for q < 0.3 nm^{-1}.

$I_1(q)$ and $I_2(q)$ are measured experimentally for, resp., the static precursor mixture and extruded bijel fragment. The structure factors $S(q, t_i)$ are calculated using the form factor of silica TMA (see SI S7).

The model has been tested by calculating $I(q, t_i)$ at various times. In these simulations, ϕ_0 equals 1 i.e. all particles in the precursor are freely dispersed. t_f was set to 611 ms i.e. the latest time recorded. Here, we first set ϕ_f = 0.2 as guess-function.

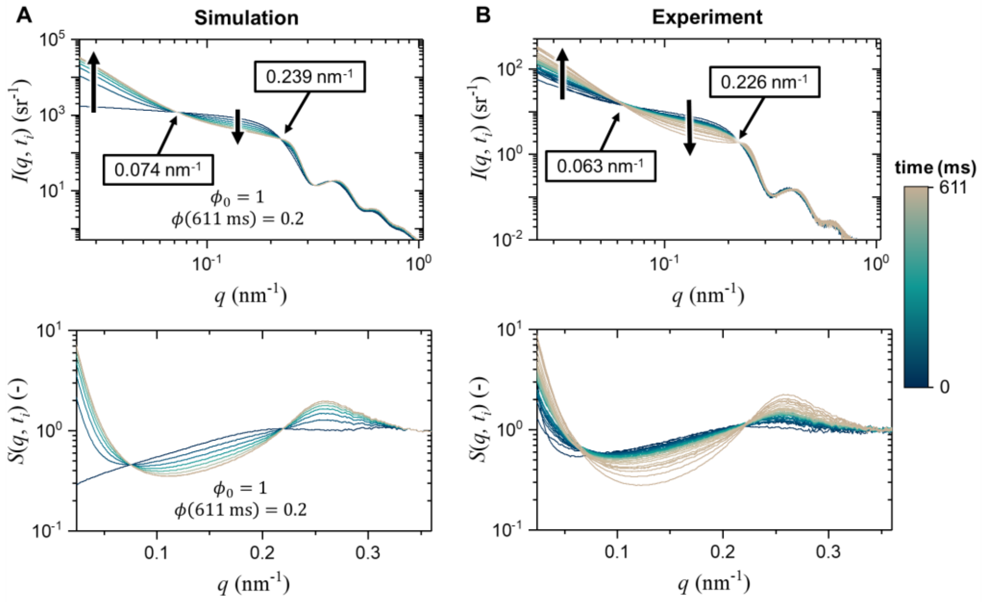

Figure S9A: $I(q, t_i)$ and $S(q, t_i)$ patterns as **A)** simulated, and **B)** experimentally measured.

Figure S9A plots the simulated and measured $I(q, t_i)$ and $S(q, t_i)$ using the described model. Both methods shows similar patterns in $I(q, t_i)$ and $S(q, t_i)$ and have two iso-scattering points near q = 0.07 nm^{-1} and q = 0.23 nm^{-1}. The patterns show that $I(q, t_i)$ and $S(q, t_i)$ remain constant for q > 0.3 nm^{-1}, decreases between the iso-scattering points and increases for q < 0.074 nm^{-1}.

Despite these similar patterns, the simulation has three main differences compared to the measurement: I) larger q-values for the iso-scattering points, II) less decrease in $I(q, t_i)$ and $S(q, t_i)$ between the iso-scattering points, and III) $S(q, 0\ \text{ms})$ decreases for q < 0.07 nm^{-1} which increases in the experiment. These differences may arise due to the value selected for ϕ_f. To study the effect of ϕ_f, the simulations were adjusted by varying ϕ_f between 0.01 and 0.5.

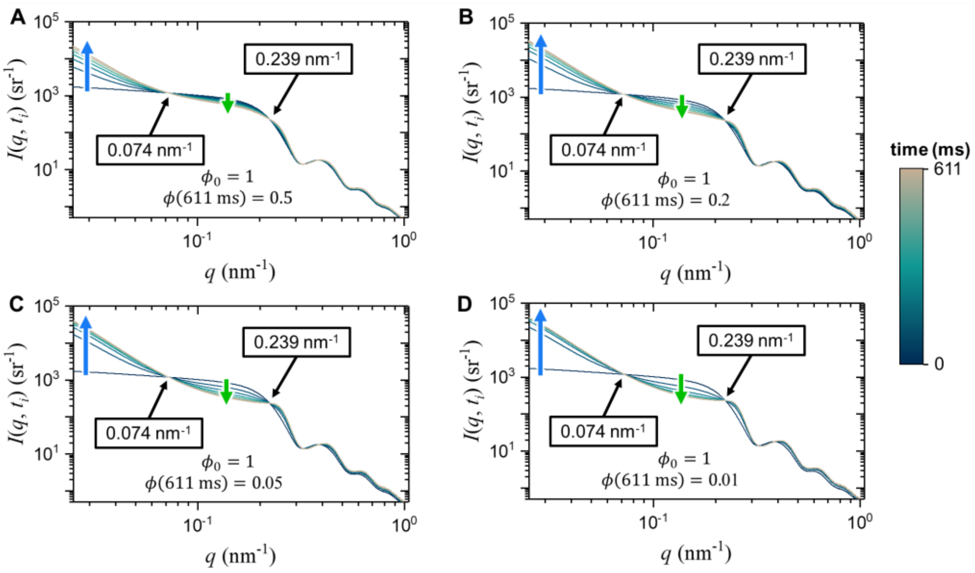

Figure S9B: Effect of ϕ_f on $I(q, t_i)$ using ϕ_0 = 1 for ϕ_f equals **A)** 0.5, **B)** 0.2, **C)** 0.05, and **D)** 0.01.

Figure S9B shows four simulated $I(q, t_i)$ patterns with various ϕ_f while keeping $\phi_0 = 1$. Interestingly, the iso-scattering points remain at q = 0.074 and q = 0.239 nm^{-1} for various ϕ_f. However, the change in $I(q, t_i)$ highly depends on ϕ_f. Lower values of ϕ_f result in a larger decrease in $I(q, t_i)$ for q ranging between the iso-scattering points as shown by the green arrows. Simultaneously, $I(q, t_i)$ increases more rapidly for q < 0.074 nm^{-1} indicated by the blue arrows. These results show that ϕ_f only affects the kinetics of the bijel formation without altering the position of the iso-scattering points and $I_{meas}(q, 0)$.

The differences in iso-scattering points and $S(q, 0)$ between the simulation and experiment, though, may originate from the differences in scattering of the precursor. In the simulation, $I_1(q)$ has been set using a static precursor which is actually flowing in the experiment. Despite these differences, no further optimization of the model has been done at this stage. This proposed model easily describes the similar pattern in both $I(q, t_i)$ and $S(q, t_i)$ compared to the experiment. Therefore, our model can describe the phase separation during bijel formation. However, the scale this model applies, has not been reported yet.

To validate the scale of the kinetic model, the normalized structure factor at q = 0.025 nm^{-1} (corresponding to clusters of 250 nm) and 0.150 nm^{-1} (individual nanoparticles) are calculated for various values of ϕ_f and ϕ_0 = 1. **Figure S9C** plots $S(q = 0.025\ \mathrm{nm}^{-1})$ and $S(q = 0.150\ \mathrm{nm}^{-1})$ as measured experimentally and simulated for various ϕ_f. It shows for q = 0.025 nm^{-1} that no distinct value for ϕ_f matches the experimental structure factors. For q = 0.150 nm^{-1}, the simulation and experiment resemble each other for $\phi_f = 0.10$. The structure factors at both scales indicate that the two-phase model simulates the kinetics on the nanoparticle-level i.e. attachment of nanoparticles on the

liquid-liquid interface. Larger structures like aggregates and pores, cannot be described by this model since other factors like vanderWaals-forces and coarsening of the domains deforms the particle scaffold.

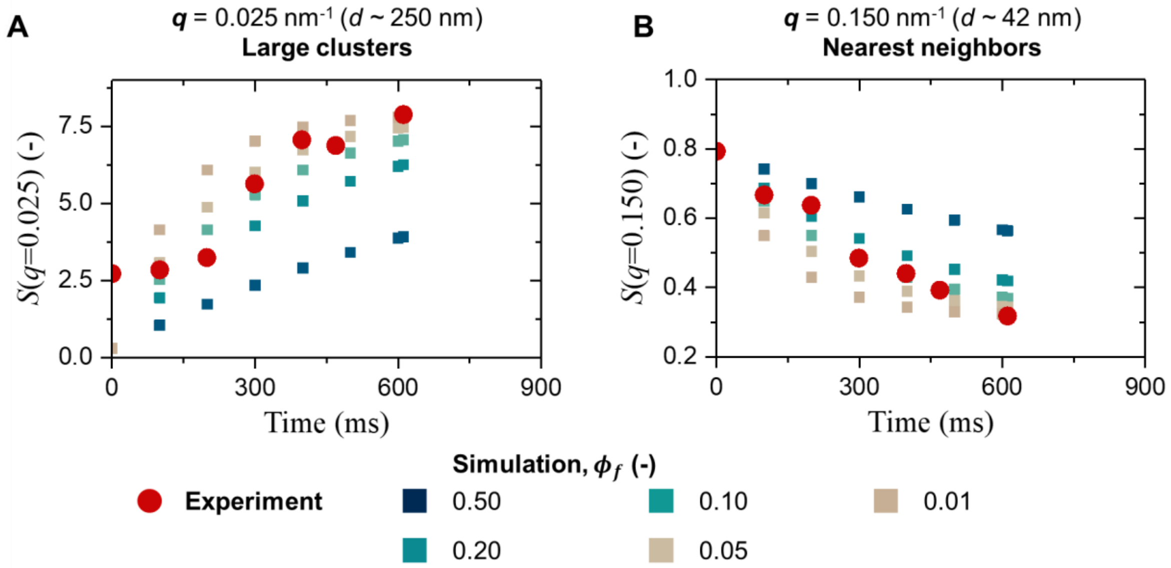

Figure S9C: Comparison between structure factors determined experimentally (circle symbols) and simulated with various values for ϕ_f (square symbols) for **A)** q = 0.025 nm^{-1} and **B)** q = 0.150 nm^{-1}.

Supporting Information 10 **Scattering intensity of flowing and static precursor**

Figure S10 shows $I(q, 0\,\text{ms})$ of the precursor mixture under static and flowing conditions. The scattering intensity for the precursor under static and flowing conditions is similar for q > 0.06 nm^{-1}, while its scattering is significantly stronger for q < 0.06 nm^{-1} under flowing conditions. This deviation may be explained by an extrusion effect which mechanism remains unclear in this study.

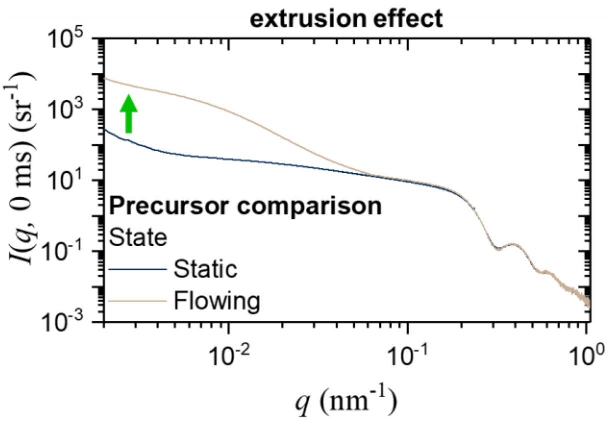

Figure S10: $I(q, 0\,\text{ms})$ of the precursor mixture as measured in (black) static capillary i.e. no flow, and (sand) flowing immediately after injection in a coaxial flow of toluene in a microfluidic device.

Supporting Information 11 Calculation of Scattering Length Densities (SLD) and their effect on the contrast in SAXS and USAXS.

To illustrate the importance of different contributions to SAXS and USAXS intensity, simple theoretical estimates were made. First of all, the typical scattering contrast was estimated as shown in **Table S11**. First of all, the real part of the refractive index $n = 1 - \delta$ of substances constituting the solution was obtained from reference [2], where δ is the refractive index decrement. Then, the scattering length density (SLD) values were calculated using

$$\rho_i = 2\pi\delta/\lambda^2$$

for several cases. Note that the exact compositions of the two liquid media, which are undergoing the spinodal phase decomposition, are time-dependent, approaching the oily phase mostly dominated by toluene and the aqueous phase, which is believed to be mostly composed of water and glycerol. In addition, silica nanoparticles with a much higher SLD, are dispersed in the system.

The scattering intensity is scaled with the square of the SLD contrast, $(\rho_i - \rho_j)^2$ between media i and j. For the liquid phases, it reaches its maximum of $(\rho_{aq} - \rho_t)^2$ at the end of the phase separation. While the liquid phases have relatively low contrast, silica nanoparticles have much higher SLD. As a result, the scattering due to silica nanoparticles exceeds the scattering intensity due to the density difference between the fluids, by a factor of about 30.

Table S11: Material properties and calculated SLD of precursor components and toluene.

	**vol-% (v/v)**	**Density (g mL^{-1})**	**δ at 12.4 keV (-)**	**SLD (10^{-4} nm^{-2})**
**Precursor**				
DEP	7.8	1.12	1.59×10^{-6}	10.0
H_2O	43.5	1.00	1.50×10^{-6}	9.44
1-propanol	38.1	0.803	1.23×10^{-6}	7.72
glycerol	10.6	1.261	1.85×10^{-6}	11.6
Total	100	0.96		$\rho_p = 9.06$
**Silica**	100	2.2	2.99×10^{-6}	$\rho_{SiO_2} = 18.8$
**Precursor + 30 wt-% silica**	87 + 13	1.124		$\rho_{p+SiO_2} = 10.3$
**Toluene**	100	0.8623	1.28×10^{-6}	$\rho_t = 8.02$
**Water+glycerol**	80 + 20	1.05		$\rho_{aq} = 9.87$

As a result, the signal in the SAXS measurements is dominated by silica nanoparticles. However, this is no longer the case in the USAXS data, which correspond to much larger spatial scales. For order-of-magnitude estimations, the following model was used.

For the USAXS regime, one can neglect details on the scale of the particle size and approximate the particle layer by a uniform layer as illustrated in the **Figure S11A**. Let us consider a liquid droplet of the radius R and the SLD ρ_2. It is covered by a silica shell with a thickness d and SLD ρ_3. This core-shell particle is then inserted in another liquid with SLD ρ_1. The scattering intensity $I(q)$ (= differential scattering cross-section) of such a core-shell particle can be calculated as

$$I(q) = (4\pi)^2 \left[(\rho_3 - \rho_1) \frac{\sin q(R+d) - q(R+d)\cos q(R+d)}{q^3} - (\rho_3 - \rho_2) \frac{\sin qR - qR\cos qR}{q^3} \right]^2$$

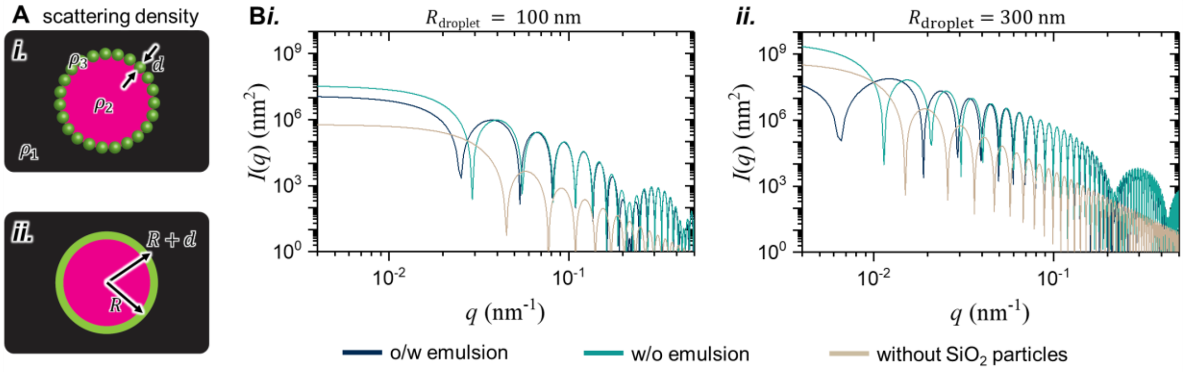

Figure S11: Scattering cross sections. **A*i*)** Schematic droplet of liquid phases 1 and 2 separated by a solid particle phase 3 with thickness d all with respective scattering length density ρ_i. **A*ii*)** Schematic dimensions of core-shell droplets with liquid radius R and droplet radius $R + d$. **B)** Scattering intensity profiles of core-shell droplets against scattering vector for droplets with radius of ***i*)** 100 nm and ***ii*)** 300 nm.

The scattering profiles $I(q)$ are presented for no silica shell (sand), water-in-oil (green), and oil-in-water droplets (blue) as shown in **Figure S11B**. For $R = 100$ nm, the scattering of a liquid droplet without the silica shell is much weaker (by a factor of 20 to 60 at the smallest q-values) than that for droplets with silica although some difference can be already seen between water-in-oil and oil-in-water droplets due to interference between the liquid-liquid and silica-liquid contrasts. For $R = 300$ nm (i.e., droplet diameter of 600 nm is about 20 times larger than the nanoparticle diameter of 29 nm), the scattering cross-section due to the liquid-liquid contrast is comparable to the scattering due to the silica shell. A much stronger interference effect between the two types of contrast can also be seen.

Finally, we discuss what the position of the broad correlation peak observed in USAXS corresponds to. Is it related to the typical distance between silica-decorated liquid-liquid interfaces? Alternatively, is it

rather determined by the characteristic distance between channels of the same type that included two silica layers? To address this issue, let us consider a 1D model consisting of periodically-arranged sets of 4 layers: watery phase – silica – oily phase – silica. The thickness of the stack is $L = L_w + d + L_o + d = L_w + L_o + 2d$. The SLD are given above. The scattering amplitude is proportional to the Fourier amplitudes of the structure

$$A_n \propto \int_{-L/2}^{L/2} e^{inq_0 x}\, \rho(x)\, dx,$$

where $q_0 = 2\pi/L$ is the main spatial frequency of the structure and n is the order of the Fourier component. By placing the origin of the x-axis in the middle of the oily layer and subtracting ρ_w from all the contrasts, one gets

$$A_n \propto \int_{-L_o/2}^{L_o/2} (\rho_o - \rho_w) \cos nq_0 x\, dx + 2\int_{L_o/2}^{L_o/2+d} \left(\rho_{SiO_2} - \rho_w\right) \cos nq_0 x\, dx$$

$$= \frac{2}{nq_0}\left[(\rho_o - \rho_w) \sin n\pi \frac{L_w}{L} + \left(\rho_{SiO_2} - \rho_w\right)\left(\sin n\pi \frac{L_w + d}{L} - \sin n\pi \frac{L_w}{L}\right)\right].$$

Finally, we assume a para-crystal model with the scattering intensity

$$I(q) \propto \sum_{\mathrm{n}} |A_n|^2 \frac{1}{\pi} \frac{nw}{(q - nq_0)^2 + (nw)^2},$$

where w is the half-width at half-maximum of the first Lorentzian diffraction peak. Note that the width of the higher-order reflections is assumed to grow with n. This assumption is different from the one usually used to describe Bragg peak broadening in a crystal powder due to the finite-size effect. The increasing peak width assumption used here corresponds to the “second-type disorder” in the terminology of Guinier[3], which corresponds to a gradual loss of the positional order. The predictions of this para-crystal model is illustrated in **Figure S11C** below.

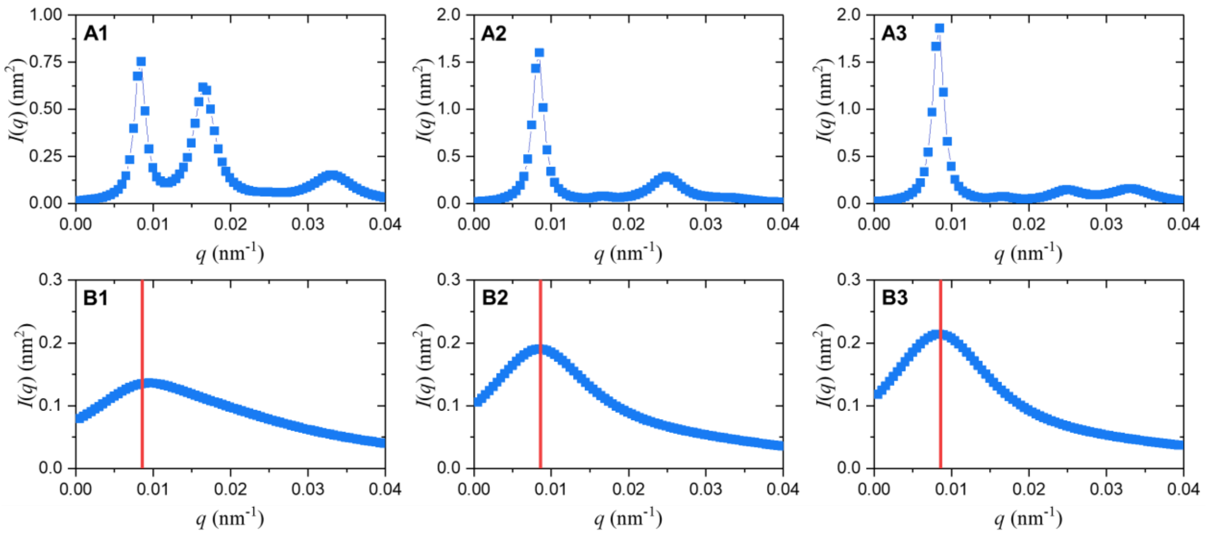

Figure S11C: Prediction of the para-crystal model for $w = 0.1\,q_0$ (top row of An panels) and $w = q_0$ (bottom row of Bn panels). $L_w = L_o$ (panels A1 and B1), $L_w = 2L_o$ (panels A2 and B2), and $L_w = 3L_o$ (panels A3 and B3). In all cases $L_w + L_o = 700$ nm. The vertical red line corresponds to $q_0 = 2\pi/L$.

To better illustrate the importance of different terms in $I(q)$, the results are first presented for $w = 0.1\,q_0$ so that the peaks can be seen separately. In panel A1, L_w is assumed to be the same as L_o so that the distance between silica layers is exactly one-half of the whole structure period $L = L_w + L_o + 2L_{SiO_2}$. In this case, the silica layers can only contribute to even diffraction peaks and the first peak at $q_0 = 2\pi/L$ originates solely from the water-oil scattering contrast. However, it is not anymore true for $L_w \neq L_o$. Moreover, one can see that the relative intensity of the peaks depends on the interference between different contributions which, in turn, depend on the exact geometry assumed in the model.

We now turn to a much more realistic situation with $w = q_0$ since the bicontinuous structure is not really periodic but can only be characterized by a characteristic sizes of the pores of two types. Here the peaks greatly overlap forming a single broad peak with the maximum close to $q_0 = 2\pi/L$. Thus, this simple estimate suggests that although silica particles have much higher contrast, the position of the peak is determined by the characteristic distance between channels of the same type that included two silica layers.

we can conclude that the scattering of the nanoparticles dominates the SAXS scattering intensity recorded. This is confirmed by the visibility of oscillations in the $I(q)$ profiles in the main text. However, in USAXS the scattering induced by the contrast between the oil- and aqueous phase start to contribute to similar extents as silica nanoparticles.

References

(1) Bird, R. B.; Steward, W. E.; Lightfoot, E. N. Flow through a Circular Tube. In *Transport Phenomena*; Wiley: New York, 1960; pp 48–56. https://doi.org/10.1136/vr.86.20.592.

(2) Henke, B. L.; Gullikson, E. M.; Davis, J. C. X-Ray Interactions: Photoabsorption, Scattering, Transmission, and Reflection at E = 50-30, 000 EV, Z = 1-92. *At. Data Nucl. Data Tables* **1993**, *54* (2), 181–342. https://doi.org/10.1006/adnd.1993.1013.

(3) Guinier, A. *X-Ray Diffraction in Crystals, Imperfect Crystals, and Amorphous Bodies ; Translated by Paul Lorrain and Dorothée Sainte-Marie Lorrain*, 1st ed.; San Francisco, 1963.
